# Alcohol Consumption and Abstention among Pregnant Japanese Women

**DOI:** 10.2188/jea.JE2007419

**Published:** 2008-08-07

**Authors:** Yuko Yamamoto, Yoshitaka Kaneita, Eise Yokoyama, Tomofumi Sone, Shinji Takemura, Kenshu Suzuki, Akiyo Kaneko, Takashi Ohida

**Affiliations:** 1 Division of Public Health, Department of Social Medicine, Nihon University School of Medicine; 2 Department of Public Health Policy, National Institute of Public Health, Japan

**Keywords:** Alcohol Drinking, Temperance, Pregnancy, Pregnant Women

## Abstract

**Background:**

In order to clarify the alcohol consumption status of pregnant women in Japan and the characteristics of pregnant women who abstained from alcohol after their pregnancy had been confirmed, a nationwide questionnaire-based study of alcohol consumption behavior was performed. We also examined the factors associated with alcohol consumption during pregnancy and abstention after the confirmation of pregnancy.

**Methods:**

After random sampling, 260 institutions participated in the survey; these were selected from a list of survey points fixed by the Japan Association of Obstetricians and Gynecologists. The study was conducted on pregnant women with confirmed pregnancies by using self-administered anonymous questionnaires during the period from February 1 through 14, 2002.

**Results:**

Alcohol consumption during pregnancy was reported in 11.1% of the study participants, and abstention after the confirmation of pregnancy, in 76.9%. Significant associations were recognized between higher education and both alcohol consumption during pregnancy and abstention after pregnancy confirmation. Furthermore, alcohol consumption was significantly associated with parity, smoking, and shorter sleep duration, whereas abstention was significantly associated with less frequent alcohol consumption and knowledge regarding the risk of alcohol consumption.

**Conclusion:**

The results clarified the factors associated with alcohol consumption during pregnancy and abstention after the confirmation of pregnancy in Japan.

## INTRODUCTION

The hypothesis that alcohol consumption during pregnancy might induce obvious abnormalities in infants was established in 1973 in a report by Jones et al.^1^ Since then, many studies have investigated the effects of alcohol consumption by pregnant women on their infants. For example, in 1986, a report revealed that maternal alcohol consumption during pregnancy played a role in mental retardation^2^ and neurobehavioral deficits^3^ observed in the infants. Furthermore, relationships between alcohol consumption and reduced fetal growth,^4^ preterm birth,^5^ and congenital malformation^6^ were reported in 1995, 2003, and 2005, respectively. Infants born to mothers with moderate to high alcohol intakes during the 1st trimester of pregnancy, i.e., weeks 3-8 of pregnancy, are often observed to have dysmorphic facial features, referred to generically as fetal alcohol syndrome.^7^ In Japan, the incidence of this syndrome is estimated to be 1 in 10,000-20,000 births.^8^ It is also reported that fetal alcohol syndrome is related to increased frequency of spontaneous abortion,^9^ decreased height and weight, increased craniofacial abnormalities,^10^^-^^12^ neurobehavioral deficits,^13^^,^^14^ and long-term effects on psychopathology, behavior, and intelligence.^15^ In addition to these studies, it was reported recently that besides alcohol consumption during pregnancy, caffeine intake and smoking are associated with childhood acute lymphoblastic leukemia and childhood acute myeloblastic leukemia.^16^^,^^17^ Backed by these reports, the US Surgeon General announced in 2005 that there was no safe amount or type of alcohol or safe time for alcohol consumption during pregnancy.^18^ A similar announcement was made by the Public Health Agency of Canada in 2007.^19^ In Japan also, starting in 2006, the Ministry of Health, Labour and Welfare set a goal of abstention during pregnancy in order to maintain and improve the level of child health care as part of a national campaign-the “21^st^ Century Healthy and Happy Family” that advocated and promoted a general framework for ensuring maternal health. Besides the “Meeting of Planning Support to Pregnant Women through Nutrition” investigated abstention during pregnancy and lactation and provided nutrition guidelines for pregnant women. It reported that alcohol consumption during the 1^st^ trimester was associated with congenital malformations and that during the 3^rd^ trimester, with mental retardation and a dysfunctional central nervous system.^20^ A large-scale study on the alcohol consumption habits of pregnant women would help to promote and develop this national campaign because few such studies have been conducted in Japan. The prevalence of alcohol consumption during pregnancy was included as a survey item in surveys on the growth of infants and preschool children that were conducted in 2000 by the Ministry of Health, Labour and Welfare.^21^ However, this survey had the following limitations: (1) the subjects were surveyed after they had given birth, and (2) the years during which the subjects were pregnant varied considerably. A more accurate survey that involves pregnant women is therefore required.

Hence, in this study, we clarified the alcohol consumption status of pregnant women and developed effective measures for dissuading them from consuming alcohol. We then examined the factors associated with alcohol consumption during pregnancy. Furthermore, by examining the characteristics of pregnant women who gave up alcohol drinking after the confirmation of pregnancy, the factors associated with abstention among women who consumed alcohol before pregnancy confirmation were identified.

## METHODS

### Subjects

We randomly selected 500 obstetric clinical institutions after stratification according to the type of institution (public hospital, private hospital, or clinic) and its location, i.e., the area block to which each institution belonged. These institutions were selected from 989 institutions nationwide that are used as fixed monitoring facilities for questionnaire surveys conducted by The Japan Association of Obstetricians and Gynecologists, which account for 10.2% of all the obstetrics and gynecology institutions in Japan. A set of the documents listed below was sent by the National Institute of Public Health to the 500 institutions: an invitation letter to the survey from the Chairman of the Association, the survey procedures, and a reply postcard indicating willingness/unwillingness to participate and the annual number of live births in the institute. Postcards were returned from 390 institutions, and these were collected by the National Institute of Public Health via the secretariat of the Japan Association of Obstetricians and Gynecologists. After excluding 110 institutions that rejected the participation, survey sheets were sent to the remaining 280 institutions. Because no pregnant woman visited the outpatient clinics in 20 institutions, the survey was eventually conducted in 260 institutions.

The study subjects were women with confirmed pregnancies who had a 2nd or subsequent consultation at one of these institutions during the period from February 1 through 14, 2002. Women having their 1st consultation during this fortnight, those whose pregnancies were not confirmed, or those who did not wish to continue the pregnancy, were excluded from the study.

### Survey

Each subject was requested to fill out a self-administered anonymous questionnaire while waiting at the institution. From each institution, all women who met the abovementioned criteria were selected as subjects. The questionnaires included a statement that the staff of the institutions would not see the completed questionnaires, which were collected in sealed envelopes. This was done in order to protect the privacy of the subjects and to obtain responses that were as candid as possible.

The number of annual live births at each institution was obtained from the institutions that agreed to participate in the survey. The institutions that replied to this question were sent 10% more questionnaires than half of their reported number of live births. The institutions distributed a total of 17,521 questionnaires to pregnant women. The institutions returned the completed questionnaires (a total of 16,528), yielding a response rate of 94.3%. Thus, approximately 6% of the subjects refused to answer the distributed questionnaire.

Identical self-administered questionnaires were used for each institution. The following survey items were included in the questionnaire: age group (10-19, 20-29, 30-39, and 40 years or older), education (graduated from junior high school, high school, or university or higher), parity, weeks of pregnancy at the time of investigation (0-11, 12-27, or 28-40 weeks or more), employment status (full-time, part-time, retired or on leave, or unemployed since before pregnancy), smoking status before pregnancy, frequency of alcohol consumption before the confirmation of pregnancy (1-2 times a week, 3-4 times a week, or daily), amount of alcohol consumed before the confirmation of pregnancy (less than one 500-mL bottle of beer, 2 bottles, 3 bottles or more per week), smoking status during pregnancy, knowledge regarding the effects of alcohol on fetuses, and whether advised about alcohol consumption.

In this study, subjects who answered “no” to the question “Before the present pregnancy was confirmed, did you regularly consume alcoholic beverages, such as beer, Japanese sake, distilled spirits, and wine?” were defined as nondrinkers before the confirmation of pregnancy, and those who answered “yes” were defined as drinkers before the confirmation of pregnancy. In addition, those who answered “no” to the question “Do you currently consume alcoholic beverages?” were defined as nondrinkers during pregnancy, and those who answered “yes” were defined as drinkers during pregnancy. The questions regarding alcohol consumption are listed in the Appendix.

### Analysis

First, the proportion of alcohol consumption before pregnancy confirmation and that during pregnancy was calculated. Mann-Whitney U test was used to examine the association of alcohol consumption during pregnancy with the age group, education, parity, weeks of pregnancy, employment status, smoking during pregnancy, and sleep duration. To examine the factors associated with alcohol consumption during pregnancy, multiple logistic regression analysis was performed, considering alcohol consumption (0: no and 1: yes) during pregnancy as a response variable. Explanatory variables were inputted into the model as covariates by using a procedure for variable selection in which all variables in a block were entered in a single step. The adjusted odds ratios and 95% confidence intervals were calculated.

Second, the proportion of abstention after pregnancy confirmation among women who drank before was calculated. The methods were the same as those used in the analyses of alcohol consumption.

In the statistical analyses, dummy variables were used for categories with 3 or more groups. A two-sided test was used, and the level of significance was set as *P* < 0.05. For statistical processing, the Statistical Package for the Social Sciences (SPSS)^®^ for Windows version 11.5 was used.

This study was approved by the ethics committee of the National Institute of Public Health.

## RESULTS

Of the 16,528 subjects, 2,289 who did not answer all the questions were excluded. Data for the remaining 14,239 subjects were analyzed as valid responses. The differences between the 2,289 excluded cases and 14,239 analyzed cases were examined using the Mann-Whitney U test (not shown as a table). It was found that women with less education, those in their 2nd or subsequent pregnancy, those employed as part-time workers, and those who smoked comprised a greater proportion of the excluded cases as compared to the other women in the study.

The age distribution of the participants is shown in Table 1, and the mean age was 29.3 years (standard deviation: 4.5 years).

**Table 1.** Characteristics of analyzed samples.

Item	n	%
Total	14239	100

Age (y)		
10-19	187	1.3
20-29	7306	51.3
30-39	6552	46.0
40+	194	1.4

Education		
Junior high school	521	3.7
High school	7946	55.8
University or higher	5772	40.5

Parity		
First	7292	51.2
Second or more	6947	48.8

Weeks of pregnancy		
0-11	1859	13.1
12-27	7251	50.9
28-40	5129	36.0

Employment status		
Full-time	2727	19.2
Part-time	978	6.9
Retired or on leave	4751	33.4
Unemployed at all times	5783	40.6

Alcohol drinking before pregnancy		
Nondrinker	7682	54.0
Drinker	6557	46.0

Alcohol drinking after pregnancy		
Nondrinker	12659	88.9
Drinker	1580	11.1

Knowledge regarding risks of alcohol drinking		
No	3868	27.2
Yes	10371	72.8

Whether advised regarding the risks of alcohol consumption during pregnancy		
No	3646	58.0
Yes	2645	42.0

Smoking during pregnancy		
Nonsmoker	12917	90.7
Smoker	1322	9.3

Sleep duration (h)		
<6	880	6.2
≥6	13359	93.8

Regarding the participants’ education, 3.7% had graduated from junior high school; 55.8%, from high school; and 40.5%, from university or higher institutions. Regarding parity, at the time of the survey, 51.2% participants were in their 1st pregnancy, and 51.2% were in their 2nd or subsequent pregnancy. Further, 74.0% participants were unemployed at the time of the survey, and the remaining 26.0% worked either full-time or part-time.

Of the 14,239 subjects, 6,557 had been drinkers before pregnancy confirmation; thus, the proportion of alcohol consumption was 46.0%. Furthermore, 1,580 women drank during pregnancy, and thus, the proportion of alcohol consumption during pregnancy was 11.1%. After this analysis, 802 women who did not answer all questions were excluded from the 6,557 women who had been drinkers before pregnancy confirmation. Of the remaining 5,755 women, 4,426 quit drinking alcohol during pregnancy, which means that the proportion of abstention was 76.9%.

The items for examining the factors associated with alcohol consumption and the results of the Mann-Whitney U test and multiple logistic regression analysis are shown in Table 2. Using multiple logistic regression analysis, we determined whether significant associations existed among the following items: age group, education, parity, weeks of pregnancy, employment status, smoking during pregnancy, and sleep duration.

**Table 2.** Factors associated with alcohol consumption during pregnancy.

				Multivariate analysis ^†^		

Item ^‡^	n	Proportion of drinkers (%)	*P* value^*^	Adjusted odds ratio	95% CI	*P* value
		
Total no. of participants	14239	11.1				

Age (y)			<0.001			
10-19	187	5.9		0.57	0.30-1.07	0.081
20-29	7306	10.0		1.00	reference	
30-39	6552	12.6		1.21	1.09-1.36	0.001
40+	194	6.7		0.58	0.33-1.02	0.058

Education			0.157			
Junior high school	521	12.3		1.13	0.85-1.50	0.410
High school	7946	10.6		1.00	reference	
University or higher	5772	11.6		1.16	1.04-1.30	0.009

Parity			<0.001			
First	7292	9.5		1.00	reference	
Second or more	6947	12.8		1.36	1.21-1.53	<0.001

Weeks of pregnancy			<0.001			
0-11	1859	6.0		0.46	0.37-0.56	<0.001
12-27	7251	12.1		1.00	reference	
28-40	5129	11.5		0.97	0.87-1.09	0.627

Employment status			0.879			
Full-time	2727	11.1		1.12	0.97-1.30	0.136
Part-time	978	13.0		1.32	1.07-1.62	0.010
Retired or on leave	4751	10.6		1.07	0.93-1.22	0.354
Unemployed since before pregnancy	5783	11.2		1.00	reference	

Smoking during pregnancy			<0.001			
Nonsmoker	12917	10.7		1.00	reference	
Smoker	1322	15.4		1.53	1.29-1.81	<0.001

Sleep duration (h)			0.006			
<6	880	14.0		1.25	1.03-1.53	0.028
≥6	13359	10.9		1.00	reference	

* : Calculated by Mann-Whitney’s U test.

† : Adjusted odds ratios and their 95% confidence intervals were calculated using multiple logistic regression analysis. The presence/absence of alcohol consumption during pregnancy was used as a response variable.

‡ : As explanatory variables, age group, education, parity, weeks of pregnancy, employment status, smoking status during pregnancy, and sleep duration were input into the model as covariates by using a procedure for variable selection in which all variables in a block are entered in a single step.

CI: confidence interval

The items examined in order to determine the factors associated with abstention and the results of Mann-Whitney U test and multiple logistic regression analysis are shown in Table 3. Significant associations were recognized using multiple logistic regression analysis among the following items: age group, education, parity, weeks of pregnancy, frequency of alcohol consumption, and knowledge regarding the risks associated with alcohol consumption. To examine the effect of multicollinearity caused by simultaneously inputting the frequency of alcohol consumption before the confirmation of pregnancy and the amount of drinking, the odds ratios when both factors had been input and when one of the factors had been excluded were examined. Because the trends of the odds ratios were similar, the effect of multicollinearity was estimated to be negligible, if at all present.

**Table3.** Factors associated with abstention after the confirmation of pregnancy.

				Multivariate analysis ^†^		

Item ^‡^	n	Proportion of abstention (%)	*P* value^*^	Adjusted odds ratio	95% CI	*P* value
		
Drinkers before pregnancy	5755	76.9				

Age (y)			<0.001			
10-19	71	87.3		1.43	0.69-2.97	0.339
20-29	2909	79.0		1.00	reference	
30-39	2702	74.2		0.91	0.79-1.04	0.161
40+	73	86.3		2.13	1.07-4.23	0.031

Education			0.032			
Junior high school	259	77.2		1.08	0.78-1.50	0.629
High school	3191	78.0		1.00	reference	
University or higher	2305	75.4		0.78	0.69-0.90	<0.001

Parity			<0.001			
First	3208	81.4		1.00	reference	
Second or more	2547	71.3		0.56	0.49-0.65	<0.001

Weeks of pregnancy			0.002			
0-11	734	86.9		2.49	1.97-3.15	<0.001
12-27	2914	74.6		1.00	reference	
28-40	2107	76.6		1.07	0.93-1.22	0.375

Employment status			0.007			
Full-time	1141	77.9		1.06	0.88-1.27	0.556
Part-time	411	74.2		0.90	0.70-1.16	0.429
Retired or on leave	2105	79.7		1.15	0.98-1.35	0.089
Unemployed since before pregnancy	2098	74.1		1.00	reference	

Frequency of drinking			<0.001			
1-2 times/wk	1111	73.9		1.00	reference	
3-4 times/wk	1282	69.4		0.74	0.61-0.90	0.003
Daily	3362	80.8		0.60	0.50-0.73	<0.001

Amount of alcohol consumed (per wk)			<0.001			
One 500-mL bottle of beer or less	1777	80.3		1.00	reference	
Two 500-mL bottles of beer	1765	78.2		1.22	0.98-1.51	0.073
Three or more 500-mL bottles of beer	2213	73.2		1.15	0.97-1.36	0.112

Knowledge regarding risks of drinking			0.008			
No	1301	74.2		1.00	reference	
Yes	4454	77.7		1.37	1.18-1.58	<0.001

Whether advised regarding the risks of alcohol consumption during pregnancy			0.504			
No	3246	77.2		1.00	reference	
Yes	2509	76.5		0.88	0.78-1.00	0.057

Smoking during pregnancy			0.042			
Nonsmoker	5075	77.3		1.00	reference	
Smoker	680	73.8		0.95	0.77-1.16	0.583

Sleep duration (h)			0.123			
<6	380	73.7		0.88	0.69-1.12	0.296
≥6	5375	77.1		1.00	reference	

* : Calculated by Mann-Whitney U test

† : Adjusted odds ratios and their 95% confidence intervals were calculated using multiple logistic regression analysis. The presence/absence of

alcohol consumption during pregnancy was used as a response variable.

‡ : As explanatory variables, age group, education, parity, weeks of pregnancy, employment status, frequency and amount of alcohol consumed before the confirmation of pregnancy, knowledge regarding the risk of alcohol consumption, advice about alcohol consumption, smoking status during pregnancy, and sleep duration were input into the model as covariates by using a procedure for variable selection in which all variables in a block are entered in a single step.

CI: confidence interval

## DISCUSSION

To examine the representativeness of the samples, the following 3 types of data were compared with regard to the attributes of the study participants and participating institutions. First, we compared the age distribution of the women in the study and that of women who gave birth in 2002; the latter was calculated from the results published in Vital Statistics of Japan, 2002. Second, we compared the percentage of births by the order of birth among the study participants and among women who gave birth in 2002; the latter were calculated from the results of Vital Statistics of Japan published by the Ministry of Health, Labour and Welfare in 2002. Third, we compared the area block-wise distributions of the institutions providing maternity services in Japan, the fixed monitoring facilities for questionnaire surveys conducted by the Japan Association of Obstetricians and Gynecologists, and the institutions that participated in this study. The results revealed that all 3 distributions were similar (data not shown).

Of the 500 institutions providing maternity services that had been randomly sampled from the survey points fixed by the Japan Association of Obstetricians and Gynecologists, 260 that eventually agreed to participate in this study were included. As mentioned earlier, their representativeness was examined and established. Therefore, this study was considered to reflect the actual status of pregnant women in Japan.

The results of this study showed that the proportion of alcohol consumption by women before and after the confirmation of pregnancy was 46.0% and 11.1%, respectively. Few large-scale studies on the alcohol consumption habits of pregnant women have been conducted overseas and in Japan. For comparison, we cite the results of some surveys performed in western countries although the definition of alcohol consumption differed among the studies (Table 4).^22^^-^^26^ Due to differences among survey methods, survey periods, and national culture, a direct comparison between the results of the surveys that were conducted in the United States or in European countries and those of the present study is not possible. However, these results can be used as references. Besides, the comparison of the results of a survey conducted in Sapporo City, Hokkaido in 1988^26^ with those of the present study revealed that the proportions of alcohol consumption both before and after pregnancy confirmation were lower in this study. One of the reasons for this difference may be regional differences. We could not find any previous studies that characterized the reasons for the higher prevalence of alcohol consumption in Sapporo. Therefore, it is difficult to explain the different results obtained in this study. In contrast, a previous study^27^ demonstrated that the prevalence of smoking among women in Hokkaido was the highest among the Japanese prefectures. Furthermore, another study^28^ and this study demonstrated that smoking was strongly associated with alcohol consumption. These findings suggest that alcohol and tobacco consumption is more prevalent among the inhabitants of Hokkaido. However, further studies on alcohol consumption among pregnant women and the regional differences in alcohol consumption are required. The prevalence of alcohol consumption during pregnancy was 18.1% according to the surveys on the growth of infants and preschool children conducted in 2000 by the Ministry of Health, Labour and Welfare. This value was higher than that revealed in the present study. Differences in the subjects and definitions of alcohol consumption may have affected the results.

**Table 4.** Comparison of references.

Reference No.	Year of examination	City	Country	Proportion of drinking before pregnancy (%)	Proportion of drinking after pregnancy (%)	n	Definition of drinking	Methods of survey
21	2002	Nationwide	Japan	Not investigated	18.1	10,008	Behavior	Self-administration
22	1993-1995	2 cities*	USA	65.6	5.2	7,489	Based on amount	Telephone interview
23	1998-1999	South Michigan	USA	Not investigated	15.1	1,131	Behavior	Face-to-face interview
24	1989	Valencia	Spain	72.7	62.7	1,004	Behavior	Face-to-face interview
25	1998-2002	Cantabria	Spain	49.5	22.7	1,510	Based on amount	Face-to-face interview
26	1998-1991	Sapporo	Japan	56.5	26.3	3,448	Behavior	Self-administration

* : Seattle, Washington and Minneapolis, Minnesota

However, the fact that 46.0% of the women consumed alcoholic beverages before the confirmation of pregnancy may suggest the importance of intervention regarding the risk of alcohol consumption in women contemplating pregnancy. Alcohol consumption by pregnant women may adversely affect their fetuses, regardless of whether or not they are aware of the pregnancy. Awareness about pregnancy varies among individuals, but most women become aware of being pregnant at an early stage. This period usually lasts up to and including the 12th week of pregnancy, during which fetuses undergo organogenesis. It has been reported that alcohol consumption during this period may have severe adverse effects on fetal development.^29^^,^^30^ A study on the associations between the consumption of a moderate amount of alcohol and infant birth weight reported that alcohol consumption before pregnancy and during the later stages (5-8 months) of pregnancy lowered the birth weight of infants.^31^ Therefore, although women become aware of their pregnancy at an early stage, alcohol consumption before pregnancy may affect their fetuses. However, a study conducted in the United States on women who were contemplating pregnancy within 1 year revealed that more than a half of them were at risk; the majority of them frequently consumed alcohol and/or smoked or did not undergo an HIV test.^32^ Although there are cultural differences between the US and Japan, this result may suggest that even if women contemplate pregnancy they may not abstain from factors that are believed to adversely affect pregnancy. A study on the issue of alcohol consumption before pregnancy confirmation was recently published in the US.^33^ It is also important to study the alcohol consumption before the confirmation of pregnancy in Japanese women.

With regard to age, the proportion of alcohol consumption during pregnancy was significantly higher, and that of abstention was significantly lower among women in their 30s, whereas the converse was true in the case of women in their 40s. These results clearly indicated that pregnant women in their 30s were at a higher risk for alcohol consumption. However, few studies have reported the relationship between the age of pregnant women and alcohol consumption, and thus further investigation of this issue is required.

The proportion of alcohol consumption during pregnancy among women who had graduated from university or received higher education was significantly higher, and the proportion of abstention was significantly lower. This result indicated that pregnant women with higher education were at higher risk for alcohol consumption during pregnancy. Similar results have also been obtained in previous studies conducted in other countries. A study conducted in 9 European countries indicated that the amount of alcohol consumed by women who had received higher education was greater than that among women with less education.^34^ A study conducted in New Zealand revealed that 41.6% women consumed alcohol during pregnancy, and that the percentage was higher in the case of women who had received higher education.^35^ However, unlike the results of this study, one study has found that higher education was one of the characteristics of women who abstained from drinking.^25^ Therefore, further careful examinations are required to clarify the association between alcohol consumption/abstention during pregnancy and education.

With regard to parity, the proportion of alcohol consumption during pregnancy was significantly higher and that of abstention was significantly lower among women who were in their 2nd or subsequent pregnancy at the time of the survey. This result suggested that experience of pregnancy may reduce wariness about alcohol consumption. The fact that the proportion of alcohol consumption was higher among multiparas than among primiparas must be one of risk factors of alcohol consumption during pregnancy. It is inferred that among multiparas, familiarization with special circumstances like pregnancy may lead to downplaying the risk of alcohol consumption during subsequent pregnancies. Health guidance for this particular group may be required in the future.

The proportion of alcohol consumption among women who were between weeks 0-11 of pregnancy was significantly low, and that of abstention was significantly high. From this result, it is assumed that women are more concerned about the health of their fetuses in the earlier stage of pregnancy, immediately after the pregnancy has been confirmed, than in the later stages. However, further examination is required regarding, for instance, the factors that are associated with the increase in the proportion of alcohol consumption in the middle or later stages of pregnancy.

Smoking during pregnancy was significantly associated with the proportion of alcohol consumption during pregnancy. This result was similar to that of a previous study.^36^ It has been reported that smoking during pregnancy increases the risk of premature birth, miscarriage, stillbirth, and placental separation.^37^^-^^42^ Fetuses are substantially affected by a combination of the risks of alcohol consumption and smoking. Particular attention should be paid to pregnant women who consume both alcohol and tobacco.

Sleep duration was also significantly associated with the proportion of alcohol consumption during pregnancy. Shorter sleep duration may suggest the existence of sleep disturbance. Alcoholic beverages are often used to induce sleep, but alcohol in fact, deteriorates the quality of sleep, thus aggravating sleep disturbance;^43^ thus, a vicious cycle is perpetuated. To resolve this problem, proper education about sleep must be promoted, and active support should be available to pregnant women to remedy sleep disturbance.

A significant association was observed between knowledge about the risk of alcohol consumption to fetuses and the proportion of abstention. The effectiveness of recognizing the risk of alcohol consumption for promoting abstention has already been reported,^44^ and this was also confirmed in the present study. With regard to the propagation of such knowledge, one report^45^ has indicated that advice from health care providers to promote abstention during pregnancy was not effective, and another^46^ found that encouraging advice from physicians did not produce the desired effect. However, because 27.2% of the participants of this study (3,868 out of 14,239) did not have the appropriate knowledge, further propagation of knowledge regarding the risk of alcohol consumption may be required in the future.

Regarding the frequency of alcohol consumption before the confirmation of pregnancy, the proportion of abstention was significantly lower among women who consumed alcohol more frequently. Thus, the continuation of alcohol consumption was influenced by the frequency, and not by the amount, of alcohol consumption. There have been several previous reports about this issue: one report concluded that alcohol consumption before the confirmation of pregnancy and at an early stage of pregnancy was one of the factors that predicted the amount of alcohol consumption during pregnancy and also suggested an association between alcohol consumption before pregnancy and that during pregnancy.^47^ Another concluded that alcohol consumption before the confirmation of pregnancy was the strongest predictor of alcohol consumption during pregnancy.^48^ Further studies are needed to clarify how alcohol consumption before the confirmation of pregnancy affects alcohol consumption during pregnancy.

This study had some limitations. First, the comparison between the analyzed and excluded cases showed that with regard to some survey items, the excluded cases exhibited tendencies similar to those shown by drinkers during pregnancy. From this, it was inferred that a large number of the excluded cases were drinkers during pregnancy, suggesting that the actual prevalence of alcohol consumption during pregnancy is higher. Second, as the questionnaire we used was self-administered, the responses were subjective. A previous report found that the validity of self-administered questionnaires was higher than that of medical records for identifying alcohol consumption status during pregnancy.^49^ However, to improve accuracy, more objective methods must be examined and introduced in our future studies, such as interview surveys by medical staff in addition to questionnaire surveys and enquiring participants’ families. Third, because this was a cross-sectional survey, which studied statuses at a specific point in time, chronological changes in alcohol consumption status from the early to later stages of pregnancy could not be examined. Fourth, factors related to family and social background, which might affect the alcohol consumption behavior of pregnant women, such as annual income, family structure, and spousal alcohol consumption behavior, were not surveyed. These limitations must be resolved in our future studies.

In conclusion, to promote abstention from alcohol during pregnancy, interventions should focus on how to motivate pregnant women to free themselves of their alcohol consumption habit, regardless of how much alcohol they consumed before the confirmation of pregnancy. We would like to emphasize the importance of this issue for promoting abstention among pregnant women.

## Appendix. Questions related to alcohol consumption.

**Table:**

1.	Before the present pregnancy was confirmed, did you regularly consume alcoholic beverages such as beer, Japanese sake, distilled spirits, and wine?
2.	(Those who answered “yes” to Question 1): How frequently did you drink? (1-2 times a week, 3-4 times a week, or daily)
3.	Before the present pregnancy was confirmed, what amount of alcoholic beverages did you consume at a time? (A 500-mL bottle of beer, 180 mL Japanese sake, 90 mL distilled spirits, and 2 glasses of wine contain approximately the same amount of alcohol).
4.	Do you currently drink alcoholic beverages?
5.	Do you have any knowledge about the effects of drinking alcoholic beverages on the fetus?
6.	For those who regularly consumed alcoholic beverages before the present pregnancy was confirmed: Were you advised to quit drinking alcohol after your pregnancy had been confirmed?
7.	(Those who answered “yes” to Question 6): Who advised you?
